# The Sensory Machinery of the Head Louse *Pediculus humanus capitis*: From the Antennae to the Brain

**DOI:** 10.3389/fphys.2019.00434

**Published:** 2019-04-18

**Authors:** Isabel Ortega Insaurralde, Sebastián Minoli, Ariel Ceferino Toloza, María Inés Picollo, Romina B. Barrozo

**Affiliations:** ^1^ Centro de Investigaciones de Plagas e Insecticidas (CIPEIN), CONICET- CITEDEF, Buenos Aires, Argentina; ^2^ Laboratorio Fisiología de Insectos, Departamento Biodiversidad y Biología Experimental (DBBE), Facultad Ciencias Exactas y Naturales, Instituto Biodiversidad y Biología Experimental y Aplicada (IBBEA, CONICET-UBA), Universidad de Buenos Aires, Buenos Aires, Argentina

**Keywords:** head louse, *Pediculus humanus capitis*, host perception, behavior, antennal lobe, sensilla

## Abstract

Insect antennae are sophisticated sensory organs, usually covered with sensory structures responsible for the detection of relevant signals of different modalities coming from the environment. Despite the relevance of the head louse *Pediculus humanus capitis* as a human parasite, the role of its antennal sensory system in the highly dependent relation established with their hosts has been barely studied. In this work, we present a functional description of the antennae of these hematophagous insects by applying different approaches, including scanning electron microscopy (SEM), anterograde antennal fluorescent backfills, and behavioral experiments with intact or differentially antennectomized lice. Results constitute a first approach to identify and describe the head louse antennal sensilla and to determine the role of the antenna in host recognition. SEM images allowed us to identify a total of 35–40 sensilla belonging to seven different morphological types that according to their external architecture are candidates to bear mechano-, thermo-, hygro-, or chemo-receptor functions. The anterograde backfills revealed a direct neural pathway to the ipsilateral antennal lobe, which includes 8–10 glomerular-like diffuse structures. In the two-choice behavioral experiments, intact lice chose scalp chemicals and warm surfaces (i.e., 32°C) and avoided wet substrates. Behavioral preferences disappeared after ablation of the different flagellomeres of their antenna, allowing us to discuss about the location and function of the different identified sensilla. This is the first study that integrates morphological and behavioral aspects of the sensory machinery of head lice involved in host perception.

## Introduction

Lice are members of the order Phthiraptera, which contains nearly 5,000 species of wingless insects. They are obligate ectoparasites, living exclusively on their warm-blooded hosts. Due to the massive infestations found in children’s heads of all around the world, the head louse *Pediculus humanus capitis* gains its sanitary and epidemiological relevance. This fact was reflected in the increasing number of studies found in the literature including toxicological, evolutionary, and genetic aspects of the biology of these hematophagous insects (Kirkness et al., 2010; Toloza et al., 2010, 2018; Olds et al., 2012; Veracx and Raoult, 2012; Bressa et al., 2015; Tovar-Corona et al., 2015; Boyd et al., 2017). However, much less information is available about how these insects exploit sensory cues released by their hosts to make appropriate feeding decisions.

Besides the eyes, the antennae of insects are the main peripheral sensory organs involved in the detection of external cues relevant to their lives, such as host odors, sexual pheromones, or refuge signals, among other. Along their surfaces, a variable number of sensilla adapted to assess different modalities of stimuli are present. Irrespectively of their form and function, all sensilla are cuticular structures that encapsulate and protect neurons, which in response to the detection of specific stimuli trigger electrical signals that travel to primary relay centers in the brain (i.e., the antennal lobes, antennal mechano-sensory and motor centers, tritocerebrum, and the gnathal ganglion), where the processing of the incoming information begins. Then, further integration at higher brain centers will allow in return a stereotyped motor behavior. As a general rule, the architecture of the sensory machinery of a given species is strongly tuned to maximize the communication with their environment, for what the functional study of their sensory structures gives the opportunity to speculate about the natural habits and preferences of the individuals.

A pioneer work of Wigglesworth (1941) showed that the body louse, *Pediculus humanus humanus,* is responsive to host odors, temperature, humidity, light, and contact cues. Later morphological studies of their antenna evinced the presence of chemo-, mechano-, and thermo-hygroreceptors (Keilin and Nuttall, 1930; Miller, 1969; Slifer and Sekhon, 1980; Steinbrecht, 1994). Recently, the first functional evidences about the role of odorant receptor (OR) genes of body and head lice were reported (Pelletier et al., 2015). Although no ORs tuned to host-related isolated compounds were found, it was shown that PhumOR2 responds to a narrow set of repellent compounds (Pelletier et al., 2015). Additionally, Crespo and Vickers (2012) found that the antennae of the chewing slender pigeon louse, *Columbicola columbae*, hold few sensilla and that the antennal sensory neurons project to aglomerular antennal lobes in the brain, suggesting a simplification of their sensory machinery.

This work aimed to gain an insight into the sensory biology of the head louse *P. humanus capitis* as a first approach to understand the sensory modalities detected by the antenna related to host perception. We investigated the external morphology of its antennae. Then, we performed anterograde antennal stainings to identify and follow neural projections up to central brain structures. Finally, we studied the effect of removing different segments of the antennae on the behavior of head lice confronted to different host stimuli (i.e., scalp chemicals, humidity, and heat).

## Materials and Methods

### Insects

Head lice *P. humanus capitis* were used throughout this study. Insects were collected by dry combing the hair of children that regularly attend to elementary schools in Buenos Aires, Argentine. Once collected, head lice were transported to the laboratory according to the protocol developed by Picollo et al. (1998), which was approved by an *ad hoc* committee belonging to the Centro de Investigaciones de Plagas e Insecticidas (CONICET-CITEDEF), Buenos Aires, Argentina. Only females were selected at the laboratory for the bioassays due to their abundance and big size. Insects were individually examined under a stereomicroscope and discarded if damaged. Then, they were transferred to an environmental chamber at 18 ± 0.5°C, 70–80 ± 1% relative humidity (RH) in the dark until the experiments. The period between collection and the start of the experiments was no longer than 2 h.

### Morphology of the Antenna: Scanning Electron Microscopy

Intact females were immersed in 70% ethanol for 24 h at 22 ± 1°C. Specimens were then mounted on aluminum stubs with double-sided sticky tape and coated with gold-palladium. The antennae were then examined under a scanning electron microscope (Carl Zeiss NTS SUPRA 40, Centro de Microscopía Avanzada, Facultad de Ciencias Exactas y Naturales, Universidad de Buenos Aires). Length and diameter of different sensory structures of the antenna from 10 individuals were measured using the software Image J (National Institutes of Health, USA, https://imagej.nih.gov/ij/).

### Neural Projections: Anterograde Antennal Backfills

Live females were ventrally glued to a double-sided taped slide leaving their antennae free. Both antennae were then cutoff between the pedicel and the first flagellomere by using dissection micro-scissors under a stereoscopic microscope, and their opened tips were immediately inserted inside the glass capillaries filled with 1% neurobiotin (Neurobiotin Tracer®, Vector Laboratories, Burlingame, USA) in 0.25 M KCl. Then, live insects were maintained inside the closed Petri dishes with a wet cotton (to assure the maintenance of a humid ambient) during 6 h to allow the neuronal tracer to diffuse through the antennal nerves. After this time, the brains were dissected in Millonig’s buffer and fixed in 4% paraformaldehyde overnight at 4°C. Then, brains were rinsed in Millonig’s buffer and dehydrated and rehydrated through an ethanol series and propylene oxide (Barrozo et al., 2009). Samples were then incubated in Oregon Green-avidin (Oregon green® 488 conjugate A6374, Molecular probes, OR, USA) with 0.2% Triton X and 1% BSA overnight at 4°C.

All preparations were cleared and mounted in Vectashield medium (Vectashield Mounting Medium®, Vector laboratories, USA), and afterward, whole mounts were optically sectioned with a laser scanning confocal microscope (Olympus FV300/BX61). Following confocal scanning of the brains, 3D reconstructions of stacks of three brains were carried out by using Reconstruct © (v1.1.0.0 by John C. Fiala).

### Antennal Excisions and Behavioral Assays

On the basis of the morphological studies carried out in section “Morphology of the Antenna: Scanning Electron Microscopy,” it was possible to distinguish three flagellomeres forming the flagellum, where most of the sensilla were found. In order to study the role of the sensilla present in the different flagellomeres in the response to host-related stimuli, excisions at different levels were carried out. In all cases, the surgery was performed 2 h before the behavioral experiments with a pair of micro-scissors on immobilized lice under a stereoscopic microscope. Individuals were randomly assigned to one of four treatments: (1) *INT*: intact animals with both complete antennae, (2) *F3-*: animals without the third flagellomeres of both antennae, (3) *F2F3-*: animals without the second and third flagellomeres of both antennae, and (4) *1ANT*: animals with one intact antenna and the other without the second and third flagellomeres. Because ablation of flagellomeres/s could affect lice behavior, *1ANT* served as a surgery control group.

The responses of lice to three host-related stimuli (i.e., scalp chemicals, humidity, and heat) were examined in two-choice walking experiments performed in an experimental room under controlled light intensity (20 ± 1 lux), constant temperature (22 ± 1°C), and ambient relative humidity (50 ± 1%). A circular arena (5.5 cm diameter) covered with a filter paper as substrate was divided into two equal zones. A particular stimulus was added at one zone of the arena, while the opposite was maintained as the corresponding control side (see below for details). In each assay, a louse was released at the center of the arena, and its spatial behavior was recorded during 5 min with the aid of a video camera (KIR-J639CE20, Sony, China) connected to a digital video recorder (DVR5104HE, Dahua Technology Co. ltd, Hangzhou, China). A minimum of 15 replicates were carried out for each experimental series. The position of the stimulus was switched between left and right zones in a pseudorandom manner in order to avoid the effect of potential unwanted spatial heterogeneities. Insects were used only once and then discarded.

Three independent experimental series were performed in which the behavioral responses of intact and antennectomized head lice to chemical, hygric, and thermal stimuli were tested. The methodology applied to present the stimuli to the lice is described later.

#### Set Up of the Chemical Stimuli

Human head samples (i.e., volatile and non-volatile molecules) were obtained following the protocol described by Ortega Insaurralde et al. (2016). This study showed that although head lice are arrested around human scalp samples, they did not show differences between the human samples of different volunteers nor with the aging of the scalp sample (i.e., 60 h-old samples are as much attractive as 0 h-old samples) (Ortega Insaurralde et al., 2016). Thus, a piece of filter paper (1 cm × 1 cm) was rubbed during 30 s against the scalp of one 30-year-old female volunteer who had not bathed or used perfumed products in the 24 h previous to the extraction. Immediately after rubbing, the filter paper was located at one side of the circular arena, while a clean filter paper was placed at the opposite side. All filter papers were handled with gloves and clean forceps to avoid skin contamination. The base of the whole circular arena was homogeneously heated at 32 ± 2°C to mimic natural host conditions. Intact (*INT*) or differentially antennectomized lice (*F3-*, *F2F3-* or *1ANT*) were individually released at the center of the arena and their behavior recorded in video.

#### Set Up of the Hygric Stimulus

To generate a hygric heterogeneity in the experimental arena, the filter paper used as a substrate of half of the circular arena was maintained dry, while the opposite half was homogeneously loaded with 100 μl of tap water using a micropipette. The base of the whole circular arena was maintained at room temperature (22 ± 1°C). Immediately after (in order to minimize water evaporation), intact (*INT*) or differentially antennectomized lice (*F3-*, *F2F3-* or *1ANT*) were individually released at the center of the arena and their behavior registered in video.

#### Set Up of the Thermal Stimulus

A thermal heterogeneity was generated by heating the floor of half of the arena with a thermostatized metallic plate, while the other half was maintained at ambient temperature. Once the thermal equilibrium was achieved, the heated side was stabilized at 32 ± 2°C and the other side at 22 ± 2°C (ambient temperature). Temperature at the floor of both zones was measured before and after each assay with a contact thermocouple (Lutron electronic, PTM-806, Taiwan) to verify the thermal stability. Intact (*INT*) or antennectomized lice (*F3-*, *F2-F3-* or *1ANT*) were individually released at the center of the arena and their behavior registered in video.

### Data Analyses and Statistical Comparisons

Behavioral outputs were quantified offline from the video films by using the software The Observer (Noldus®). The preference of head lice to stay at each side of the arena was quantified as the time spent in each zone for each individual. The percentage of time at the stimulus side was calculated as a measure of its preference. Each bar in the figures represents the mean time spent at the stimulus side and the standard errors for each group of insects.

The total walking time was registered for each individual. The percentage of the experimental time walking was calculated as a measure of the activation elicited by the stimulus. Each bar in the figures represents the mean active time and the standard errors for each group of insects.

Statistical differences among groups (i.e., *INT*, *F3-*, *F2-F3-*, and *1ANT*) for both variables (i.e., preference and activity) were assessed by means of one-way ANOVAs followed by Tukey’s HSD comparisons, after checking assumptions of homoscedasticity and normality of data. Analyses were carried out by using the statistical package R (v.3.3.1) (R Development Core Team, 2016).

## Results

### External Morphology of Antenna and Sensilla

**Figure 1:**
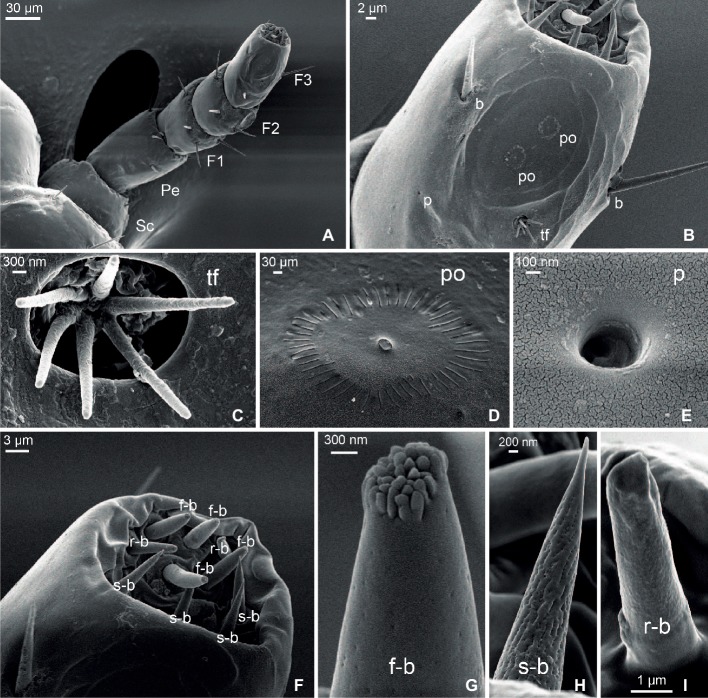
Scanning electron micrographs of the antenna of head lice. **(A)** General view of the whole antenna constituted by scapus (Sc), pedicellum (Pe), and a composed flagellum with three flagellomeres (F1, F2, and F3). **(B)** Detail of F3 showing one tuft organ (tf), two pore organs (po), two bristles (b), and one single pore (p). **(C)** Detail of a tuft organ. **(D)** Detail of a pore organ. **(E)** Detail of a single pore. **(F)** Magnification of the distal end of F3 showing three types of basiconica sensilla: finger-like (f-b), sharp-end (s-b), and round-end (r-b). **(G)** Detail of a finger-like basiconica sensillum. **(H)** Detail of a sharp-end basiconica sensillum. **(I)** Detail of a round-end basiconica sensillum.

SEM inspection allowed us to describe the external morphology of the antenna of the head louse through the characterization of the sensilla. From the base to the tip, the antenna was composed by the scapus, the pedicellum, and the composed flagellum, with a total length of 295.15 ± 15.12 μm (Figure 1A). The average length for the scapus was 53.32 ± 6.15 μm; for the pedicellum, 78.20 ± 5.54 μm; and for the flagellum, 150.18 ± 9.07 μm. The flagellum was subdivided into three sub-segments or flagellomeres, from proximal to distal named F1, F2, and F3, each one with a mean length of 50.3 ± 5.1, 41.74 ± 3.14, and 58.16 ± 4.13 μm, respectively.

Seven types of sensory structures were identified along the antenna of the head louse (Figures 1B–I): bristles, tuft organs, pore organs, single pore, and three morphs of sensilla basiconica: finger-like basiconica, sharp-end basiconica, and round-end basiconica.

Bristles were found in all segments of the antennae (Figures 1A,B). They showed a well-developed socket at the base and became constricted at the distal end. No pores were observed along their surface. The scapus, the pedicellum, and the F1 only presented bristles. Three bristles were identified on the scapus, and 5–7 on the pedicellum, all of them being similar in length: 16.46 ± 1.02 μm. Longer bristles were found at the flagellum than at the scapus and pedicellum. F1 and F2 bore from five to six bristles each of 25.56 ± 2.24 and 26.98 ± 1.67 μm of mean length, respectively. F3 only exhibited three bristles of 22.39 ± 2.69 μm of mean length.

Two tuft organs were identified, one located at the dorso-lateral side of F2 and the other at the dorso-lateral side of F3 (Figures 1B,C). Each tuft organ consisted of a deep and circular pit (3.54 ± 0.21 μm diameter) from which six pegs emerged and each with a mean length of 3.31 ± 0.18 μm.

Two pore organs centered in an oval and shallow depression (29.30 ± 2.40 μm diameter) were present on the distal dorso-lateral side of F3 (Figures 1B,D). Each pore organ exhibited a sun-like shape, bearing a central plate (0.32 ± 0.02 μm diameter) surrounded by 49 ± 1 grooves of a mean length of 0.73 ± 0.04 μm.

A single pore (Figures 1B,E) of 0.49 ± 0.03 μm diameter was located at the dorso-medial side of F3 and next to the tuft and pore organs.

The distal end of F3 exhibited different types of sensilla basiconica (Figure 1F). Among them, 10 sensilla of three morphological types were identified: four finger-like, four sharp-end, and two rounded-end ones. The finger-like basiconica (8.13 ± 0.95 μm long) were characterized by the presence of numerous short pegs at their tip and by the presence of pores all along their surface (Figure 1G). The sharp-end basiconica (9.25 ± 0.03 μm) also exhibited multiple pores uniformly distributed along the cuticle, although these sensilla had a fine and pointed end (Figure 1H). Finally, the rounded-end basiconica (6.56 ± 0.16 μm long) presented a unique pore at the tip (Figure 1I).

### Neural Projections of Antennal Sensilla

**Figure 2:**
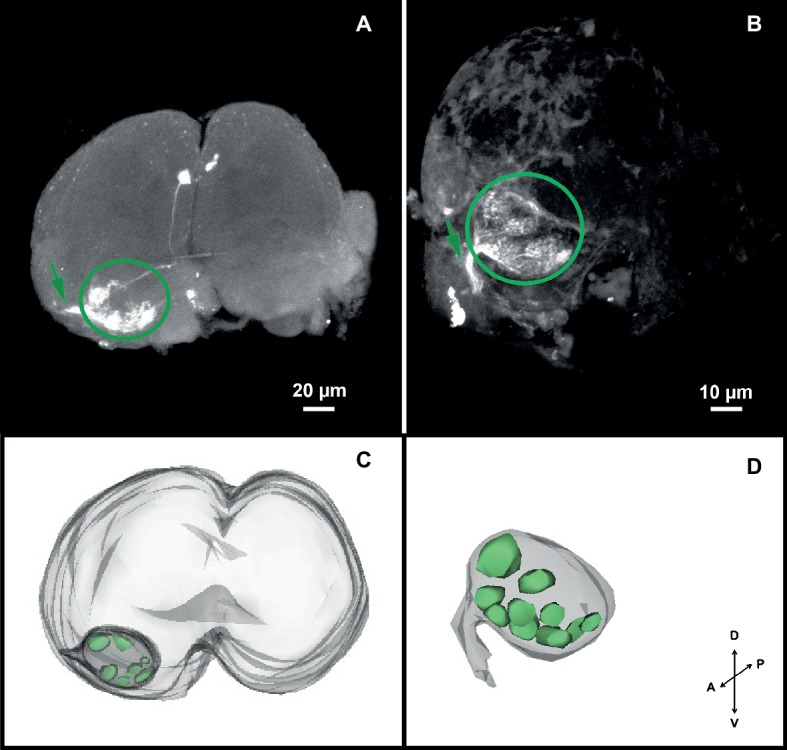
Antennal projections to the brain and antennal lobe organization of head lice. **(A**,**B)** Anterior views of the brain, showing neurobiotin antennal backfills. An antennal nerve (arrow) reaches the brain antero-ventrally and innervates the ipsilateral antennal lobe (circle), where diffuse globular structures were identified. **(C**,**D)** 3D reconstruction of the AL where 8–10 glomerular-like structures were evinced. Orientation bars: *D*, dorsal; *V*, ventral; *P*, posterior; *A*, anterior.

The confocal scanning of brains allowed us to calculate the dimensions of the head louse brain (excluding the optic lobes): 246.25 ± 11.19 μm wide, 185 ± 2 μm height. Coming from the antennae, an ipsilateral neural track (i.e., the antennal nerve: AN) reached the brain antero-ventrally and innervated a rounded-shape neuropil, likely the antennal lobe (AL) (Figures 2A,B). Each AL was situated ventrally and close to the esophageal connectives when observed from an anterior view (Figures 2A,B). The ALs measured 54.17 ± 2.46 μm wide and 38.16 ± 1.74 μm height. Inside ALs, diffuse glomerular arrangements were recognized (Figures 2A,B). 3D reconstructions (Figures 2C,D) revealed about 8–10 glomerular-like structures. Three different individuals were used for 3D reconstructions of the AL. All analyzed samples about the same glomerular structures, in number and positions, were identified. In a single neurobiotin preparation, we found a neural projection to the medial ipsilateral protocerebrum (Figure 2A).

### Behavioral Responses to Host Cues

#### Antennal Flagellomeres Involved in the Response to Human Scalp Chemicals

**Figure 3:**
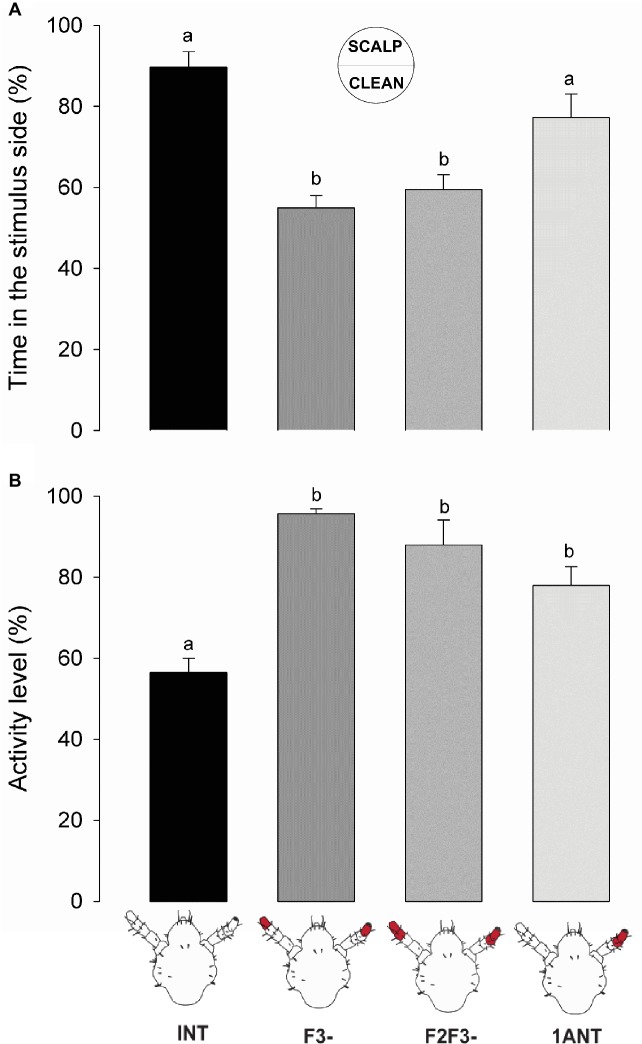
Behavioral responses of intact and antennectomized head lice to scalp chemicals. **(A)** Preference of intact and antennectomized lice in a two-choice arena, measured as the percentage of time spent at the scalp chemical side. Intact insects (*INT)* and those with only one antenna ablated (*1ANT)* spent significantly more time at the scalp chemicals side of the arena than those with F3 (*F3-*) or F2 and F3 (*F2F3-*) ablated. **(B)** Activity levels of intact and antennectomized head lice, measured as the percentage of the experimental time they remained active over the arena. *INT* lice were significantly less active than all antennectomized ones (*F3-*, *F2F3-*, and *1ANT*). Each column represents the mean of 15 insects. Different letters indicate significant differences (one-way ANOVA, *p* < 0.05).

Insects both with intact antennae (*INT*) and with only one intact antenna (*1ANT*) remained significantly more time than the antennectomized groups *F3-* and *F2F3-* at the scalp chemical side of the arena (Figure 3A, one-way ANOVA, *F* = 15.76, *df* = 3, *p* = 1.74e-07, Tukey’s HSD comparisons: *INT* vs. *F3-*, *p* < 0.001; *INT* vs. *F2F3-*, *p* < 0.01; *1ANT* vs. *F3-*, *p* < 0.001; and *1ANT* vs. *F2F3-*, *p* = 0.02). These results suggest that the absence of the flagellomere F3 is enough to cause the loss of preference in these insects.

Besides, scalp chemicals are known to exert an arresting effect on head lice (Ortega Insaurralde et al., 2016). Our results show that *INT* lice exhibited a significant lower activity level than all the other groups (Figure 3B, one-way ANOVA, *F* = 16.7, *df* = 3, *p* < 0.001, Tukey’s HSD comparisons: *INT* vs. *1ANT*, *p* = 0.0055; *INT* vs. *F3-*, *p* < 0.001; and *INT* vs. *F2F3-*, *p* < 0.01), suggesting that all the chemosensory machineries are needed to evoke an arrestment around scalp compounds (i.e., volatiles and non-volatiles).

Altogether, these results evince that when the antennae of lice were symmetrically cutoff (i.e., *F3-* and *F2F3-*) both the preference and the arrestment for the stimulus side disappeared. Intermediate results were observed in *1ANT*, where lice displayed a similar preference for the scalp chemicals as *INT* animals, but they were not arrested by such chemicals. Besides, sensory structures present in the F3 seem to be necessary and enough to perceive host chemical stimuli.

#### Antennal Flagellomeres Involved in Response to Humid Substrates

**Figure 4:**
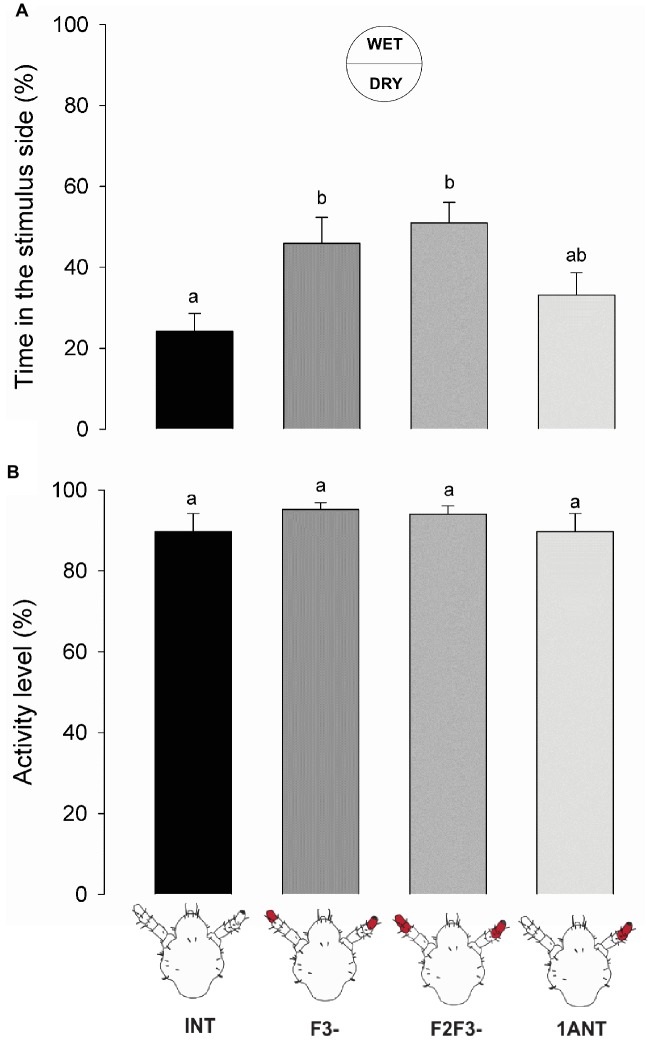
Behavioral responses of intact and antennectomized head lice to a humid substrate. **(A)** Hygric preference of intact and antennectomized lice in a two-choice arena, measured as the percentage of the time spent at the wet side. *INT* lice spent less time at the humid side of the arena than the symmetrically antennectomized ones (*F3-* and *F2F3-*). Lice with *1ANT* displayed intermediate hygric avoidance. **(B)** Activity levels of intact and antennectomized head lice, measured as the percentage of the experimental time they remained active over the arena. All groups displayed similar activity levels. Each column represents the mean of 15 insects. Different letters indicate significant differences (one-way ANOVA, *p* < 0.05).

Lice with intact antennae (*INT*) remained significantly less time at the wet side of the arena than the symmetrically antennectomized insects (*F3-* and *F2-F3-*) (Figure 4A, one-way ANOVA, *F* = 5.047, *df* = 3, *p* = 0.00364, Tukey’s HSD comparisons: *INT* vs. *F3-, p* = 0.032; and *INT* vs. *F2F3-, p* = 0.005). Insects with only one antenna (*1ANT*) showed an intermediate hygric avoidance behavior, although they showed no significant difference with the other experimental groups (Tukey’s HSD comparison, *1ANT* vs. *INT*, *p* = 0.644; *1ANT* vs. *F3-*, *p* = 0.356; and *1ANT* vs. *F2F3-*, *p* = 0.104).

On the other hand, the activity levels of lice from different experimental groups did not differ significantly (Figure 4B, one-way ANOVA, *F* = 1.451, *df* = 3, *p* = 0.238), suggesting that humidity generates a spatial aversion but not a kinetic modulation. Additionally, it confirms that ablation of antennal segments had no effects on the normal locomotion of lice.

Similar to the previous series, ablation of F3 and/or F2 and F3 caused a significant loss of the aversive response to the wet zone of the arena suggesting that F3 probably contains the sensory structures involved in humidity detection.

#### Antennal Flagellomeres Involved in Response to Heat

**Figure 5:**
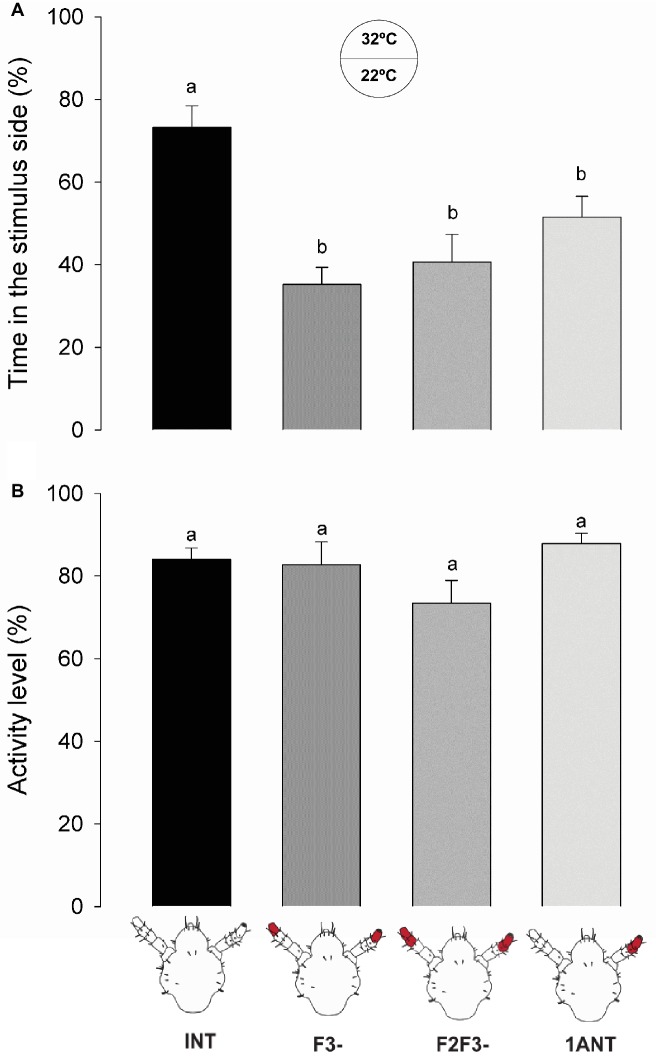
Behavioral responses of intact and antennectomized head lice to a warm substrate. **(A)** Thermal preference of intact and antennectomized lice in a two-choice arena, measured as the percentage of the time spent at the heated (32°C) side. *INT* lice spent significantly more time in the warmer side of the arena than all antennectomized ones (*F3-*, *F2F3-*, and *1ANT*). **(B)** Activity levels of intact and antennectomized head lice, measured as the percentage of the experimental time they remained active over the arena. All groups displayed similar activity levels. Each column represents the mean of 15 insects. Different letters indicate significant differences (one-way ANOVA, *p* < 0.05).

The warmer zone of the arena was preferred for head lice with intact antennae (*INT*) in relation to all the antennectomized insects (i.e.*, F3-, F2-F3-*, and *1ANT;* Figure 5A, one-way ANOVA, *F* = 9.819, *df* = 3, *p* < 0.001, Tukey’s HSD comparisons: *INT* vs. *1ANT*, *p* = 0.029; *INT* vs. *F3-*, *p* < 0.001; and *INT* vs. *F2F3-*, *p* < 0.001). The absence of F3 in lice was enough to cause the observed loss of preference for the warmer side of the arena.

No significant differences between the activity levels of the different groups of insects were observed (one-way ANOVA, *F* = 2.384, *df* = 3, *p* = 0.075, Figure 5B), suggesting that surgery did not affect the normal locomotion of insects.

These results suggest that thermo-receptors present in the F3 might be responsible for the thermal preference of head lice and that both antennae are necessary.

## Discussion

This work constitutes the first approach in the functional study of the role of the antennae of head lice in the detection and perception of environmental and/or host-related stimuli. As it happens with most animals that develop parasitic lifestyles, a relatively simple and closely host-tuned sensory system was found in *P. humanus capitis*. However, although its high epidemiological relevance as a human parasite, no morphological or functional studies of its sensory system were available in the literature before this work.

### Morphology of the Antennae

According to our description based on the SEM photographs, the overall sensory scheme of the human head louse antenna is represented by 35–40 sensilla belonging to seven different morphological types. In comparison to other blood-sucking insects (Barrozo et al., 2017), lice present a relatively low number of sensory structures on their antennae. Chapman (1982) proposed that insects living in environments where relevant stimuli doses are particularly high (like it happens in head lice walking over the human scalp) would probably have a relative low number of sensilla. This particular sensory condition may provide low sensibility, but in turn, it would offer a simplified process of integration in the central system. Another hypothesis that could explain the narrow sensorial machinery would be that a low number of sensilla could be an adaptation to prevent dehydration (Kristoffersen et al., 2008), and since human lice in general are prone to die quickly off-host due to water unbalance (Nuttall, 1919; Wigglesworth, 1941; Buxton, 1946), this could be the case.

Head louse antennae and sensilla showed to be similar to those of other Phthiraptera members described before (Miller, 1969; Ubelaker et al., 1973; Slifer and Sekhon, 1980; Baker and Chandrapatya, 1992). Along all the suborders of Phthiraptera (i.e., Anoplura, Ischnocera, Amblycera, and Rhynchophthirina), the morphology of the antennae is quite similar and conserved, characterizing the concentration of sensilla in the distal end (Baker and Chandrapatya, 1992; Soler Cruz and Martín Mateo, 1998). The head lice (*P. humanus capitis*) and body lice (*P. humanus humanus*), being closely related species, present quite comparable sensory structures. For example, Steinbrecht (1994) found tuft organs in the body lice, concluding after an exhaustive study that these structures represent thermo/hygroreceptors. Similarly, we identified tuft organs in the F2 and F3 of the head lice antennae. Steinbrecht (1994) also identified pore organs in the body lice and suggested that they could be bimodal olfactory-thermoreceptors. Here, two pore organs in the F3 of head lice were found. Besides, in other members of Phthiraptera such as livestock lice *Damalinia forficula, D. lipeuroides,* and *D. reduncae* (Soler Cruz and Martín Mateo, 1998), a single pore sensillum was observed; same structure was found and described here for the first time in the head lice. This sensillum type is similar to coeloconica sensilla identified in the other blood-sucking insects such as the kissing bugs *Triatoma infestans* (Bernard, 1974) and *Rhodnius prolixus* (Catalá and Schofield, 1994) or the bed bug *Cimex lectularius* (Steinbrecht and Muller, 1976). McIver and Siemicki (1985) assumed that these coeloconica sensilla might function as thermo-hygroreceptors in kissing bugs.

On the other hand, the pegs of the apex of the antennae of body lice were previously postulated as chemoreceptors (Wigglesworth, 1941; Slifer and Sekhon, 1980). In our work, we showed for the first time the fine structure of three morphs of these apical pegs, named basiconica. Based on their external morphology, the finger-like and the sharp-end basiconica seemed to be olfactory sensilla, as the cuticle presents uniformly distributed pores. The protrusions observed at the tip of the finger-like basiconica could increase the exposed surface to detect odors from the environment. Conversely, the round-end basiconica presented no pores along its surface but a single pore at the tip, suggesting a gustatory role or contact chemoreception.

Overall in the head louse antennae and purely based on sensilla morphology and similarities with sensory structures of other insects, we hypothesize about their function: bristles as mechanoreceptors, tuft organs as thermo-hygroreceptors, pore organs as chemo- and thermo-receptors, the single pore as thermo/hygroreceptors, finger-like and sharp-end basiconica as olfactory chemoreceptors, and rounded-end basiconica as contact chemoreceptors. Future functional studies like single sensillum electrophysiological recordings will further complement and confirm our hypothesis.

### Antennal Projections to the Brain

Sensory afferents originating from each antenna entered through antennal nerves and innervated ipsilaterally the lateral and anteroventral region of the head louse brain. Such antennal arborizations ended in the first olfactory integration neuropil: the antennal lobe (AL), without bypassing this neuropil. Even if the glomerular organization of the ALs was quite diffuse, we could identify around 8–10 glomerular structures. These results contrast with the aglomerular ALs previously reported for a closely related species, the pigeon lice *C. columbae* (Phthiraptera: Ischnocera) (Crespo and Vickers, 2012). On the opposite side, head lice showed a rather simple AL compared to other insects, such as dipterans with around 50–70 glomeruli (Laissue et al., 1999; Fishilevich and Vosshall, 2005; Ignell et al., 2005; Riabinina et al., 2016; Lin et al., 2018, etc.), bees with 160–200 (Arnold et al., 1985; Galizia et al., 1999; Roselino et al., 2015, etc.), 60–80 for moths (Masante-Roca et al., 2005; Couton et al., 2009; Zhao et al., 2016, etc.), or even some ants bearing up to 400 glomeruli (Zube and Rössler, 2008; Stieb et al., 2011, etc.). The relatively low number of glomeruli of head lice is probably the result of their life style: they are strict specialist organisms that live their entire lives on the head of human beings, which offer food, refuge, and place to lay eggs. Thus, relevant odors must be only those released by their hosts. Besides and supporting these data, the analysis of the genome of lice evinced a rather small repertoire of chemosensory-related genes (Kirkness et al., 2010). On the contrary, other blood feeders have a broader array of potential hosts than head lice. This fact likely implies more complex sensory systems tuned to perceive a major diversity of host cues. For example, it was shown for kissing bugs and mosquitoes that they use carbon dioxide and a diversity of host-emitted odors as signaling cues to find a vertebrate host (Guerenstein and Lazzari, 2009; Takken and Verhulst, 2013). About 22 glomeruli were identified in the ALs of the kissing bug *Rhodnius prolixus* (Barrozo et al., 2009) and about 50–60 in the mosquitoes *Aedes aegypti* and *Anopheles gambiae* (Ignell et al., 2005; Ghaninia et al., 2007). Taking into consideration the multiple studies on insects of different orders, the less developed ALs are most likely to be a convergent adaptation to a similar lifestyle and specific ecological and ethological requirements rather than an intrinsic feature of a given taxon (Kollman et al., 2016). The possession of few or non-defined AL glomeruli would not be direct indicators of absence or poor sensitivity to host-related cues. In different insects, conspicuous olfactory responsiveness to relevant odors was found even if they present a rather simple or aglomerular antennal lobes, e.g., head lice (this work), the pigeon louse *C. columbae* (Crespo and Vickers, 2012), but also the dragonfly *Libellula depressa* (Paleaoptera) (Rebora et al., 2013) and the psyllid *Trioza apicalis* (Homoptera) (Kristoffersen et al., 2008).

### Role of the Antenna in Host Detection

In a pioneer work, Wigglesworth (1941) showed through behavioral assays in a two-choice arena that the body louse *P. humanus humanus* is repelled by certain odors and avoids humid regions. However, the avoidance behavior stopped once the antennae were covered with cellulose paint. Later, Mumcuoglu et al. (1986) and Peock and Maunder (1993) observed that the aggregation behavior to feces of body lice and the repellency to piperonal disappeared after partial antennectomy. In the head louse *P. humanus capitis*, Ortega Insaurralde et al. (2016) observed an arrestant effect of human scalp chemicals in a two-choice arena. In the same species, Galassi et al. (2018) showed an oriented response to host odors in a T-tube olfactometer with intact antennae. In the present work, we show that the removal of the distal end flagellomere (i.e., *F3-*) of both antennae abolished this preference, suggesting that sensilla present in this segment are probably involved in host perception. F3 bears multiporous sensilla, several basiconica (i.e., finger-like and sharp-end sensilla), and pore organs, all of them being potential olfactory structures. However, we cannot discard that uniporous round-end basiconica sensilla (i.e., with potential contact chemoreceptive function) might be involved in the detection of non-volatile components present in human scalp samples. Therefore, one or both chemosensory modalities, olfaction and gustation, could guide louse behavior in our set-up. Although insects with only one intact antenna preferred the side of the arena added with scalp samples, arrestment or decrease in the activity levels only occurred when the two antennae were intact. Possibly a reduced antennal input, due to the excision of F2 and F3 of one antenna, would not be enough to trigger significant insects’ arrestment.

Head lice with intact antennae showed aversiveness to humid or wet surfaces. However, the removal of F3 significantly diluted this behavior. Water balance is a matter of importance among insects, and in fact, some terrestrial insects are prone to conserve and “grab” water from the environment under different adaptation (Hadley, 1994). However, when head lice are over a host, they feed several times a day with large intakes of water involved in every blood meal. Consequently, if a head louse has parasitized a host, water would not be a limiting resource. But, in general, excessive humidity in the environment facilitates fungi proliferation, sometimes with deleterious effects for insects, so that different species developed distinct limits of tolerance for humid environments, according to their physiology and habitat adaptations (Guarneri et al., 2002; Rowley and Hanson, 2007). Our results showed that the sensilla involved in moistness detection in head lice are located in F3. Previous works proposed the tuft organs as responsible for humidity sensing in the body lice (Steinbrecht, 1994).

Finally, only head lice with both intact antennae chose to spend more time in the warmer zone of the arena. Heat is among the most relevant stimuli used by hematophagous insects to find their warm-blooded hosts (Lazzari, 2009). At the same time, ambient temperature is known to depict the spatial and geographical distribution of most animals in earth. However, up to now, no information was available about the thermal preference of the head louse. It seems that the response to thermal stimulus depends exclusively on the bilateral input of both antennae, since the preference for the thermal stimulus disappeared in insects with only one antenna *(1ANT*). This response also faded when F3 and both F2 and F3 were ablated. The candidate sensilla to evaluate the thermal information can be the tuft organs, the pore organs, and the single pore sensillum, all three present in the F3.

Overall, our work provides new information about the sensory physiology of head lice including the structures involved in stimuli detection and processing as well as the behavior displayed in response to host-associated stimuli. Future studies should be focused on the verification of the function of each antennal sensillum type by means of electrophysiological recordings, by anterograde labeling of single sensilla, and by the study of sensory structures present in other parts of their body, such as legs and mouthparts.

## Author Contributions

IO and RB carried out the experiments. RB, SM, AT, and MP designed the experiments and provided the financial support. IO, RB, and SM wrote the manuscript. RB, SM, AT, and MP critically revised the manuscript.

### Conflict of Interest Statement

The authors declare that the research was conducted in the absence of any commercial or financial relationships that could be construed as a potential conflict of interest.
